# Exploring variations in Certificate of Vision Impairment (CVI) with geographical locations, deprivation indices, density of optometry practices and number of older people living in Mid and South Essex: an analysis of publicly available data

**DOI:** 10.1038/s41433-026-04627-6

**Published:** 2026-06-27

**Authors:** Shahina Pardhan, Rumalie Wijewicrama, Boye Tayo, Peter Scolding, Anita Pereira, Jayne Mason, Sanjiv Ahluwalia, Mapa Prabhath Piyasena

**Affiliations:** 1 Vision and Eye Research Institute, School of Medicine, Anglia Ruskin University, Young Street, Cambridge United Kingdom; 2 Centre for Inclusive Community Eye Health, School of Medicine, Anglia Ruskin University, Young Street, Cambridge United Kingdom; 3 Mid and South Essex Integrated Care System, NHS England, Basildon, Essex United Kingdom; 4 Primary Care Research Centre, School of Medicine, Faculty of Health, Medicine & Social Care, Anglia Ruskin University, Cambridge, United Kingdom

**Keywords:** Public health, Scientific community

## Abstract

**Background:**

Examining factors that contribute to Certification of Vision Impairment (CVI) is important to enable an overview of progression, plan policies and distribution eye care services.

**Objectives:**

To investigate associations between CVI, rate of change of CVI over a two-year period (2021–2023), percentage of people living in the 20% most deprived areas of Mid and South Essex (MSE), density of optometry practices and population of older people (>60 years) living in five sub-Integrated Care Board locations [Sub-ICB 1–5]) within MSE.

**Methods:**

Data were obtained from ICB databases and publicly available sources. Descriptive statistics were summarised to present data, and associations were explored using Spearman’s rank correlation.

**Results:**

CVI increased over the 2021–2023 period with age-related macular degeneration being the most common cause. In 2023, different Sub-ICB areas showed varying burden with Sub-ICB-2 having the lowest number of CVI (29.2 per 100,000) while Sub-ICB-4 and Sub-ICB-5 showing the highest (75.3–76.3 per 100,000). Sub-ICB-4 also had the highest percentage of older people (>60 years). Strong positive association (ρ = 0.90, *p* = 0.0374) was found between rate of change of CVI and deprivation. Moderate positive associations between CVI with density of optometry practices (ρ = 0.60, *p* = 0.285); and the percentage of people over the age of 60 years (ρ = 0.50, *p* = 0.391).

**Conclusions:**

CVI increases in areas with older population, and also in those living in 20% most deprived areas, highlighting the potential link between socio-economic factors and vision impairment. These factors are important for targeting interventions in specific areas to reduce the risk of blindness in MSE.

## Introduction

Vision impairment is a critical public health issue that affects quality of life [1] and has a major impact on economic productivity [2]. Worldwide in 2020, 43.3 million individuals were estimated to be blind; 295 million with moderate and severe vision impairment and 258 million with mild vision impairment [3, 4].

In the United Kingdom, individuals can become certified (i.e., Certificate of Vision Impairment [CVI]) as sight impaired (formerly referred to as partially sighted) or severely sight impaired (previously referred to as blind) [5]. Analysing annual trends in CVI certification would be important for the development of strategies and policies for eyecare allocation, and planning of the distribution of resources and organisation of eye care services [6, 7]. According to National Institute for Health and Care Excellence Serious Eye Disorders, Quality Statement number 6: “Certificate of Vision Impairment” [8], people with serious eye disorders should receive a CVI as soon they are eligible. The CVI database provides vital epidemiological data on the prevalence and severity of vision impairment at local and national levels [9]. The National Health Services (NHS) in England provides funding for free eye examinations through General Ophthalmic Services (GOS-1) for children under the age of 16 years, students in full-time education (16–18 years), everyone over the age of 60 years, individuals on low income, and individuals with eye diseases or those at risk of developing them through family history of glaucoma [10].

Over two million people in the UK are reported to be living with sight loss, of which 320,000 are registered as blind or partially sighted [11]. The majority (80%) of these are 65 years of age or older, with 60% over 75 years of age [11]. Recent data from England demonstrate a progressive increase in the rate of CVI, from 41 per 100,000 population in 2017/2018 to 43.5 per 100,000 in 2023/2024 [6, 12]. The primary causes of vision impairment include age-related macular degeneration (AMD), glaucoma, and diabetic eye disease [11, 12].

Geographical variations of CVI exist across England. Over 15 years ago, Malik et al. [13] reported 11-fold variation in CVI per 100,000 among the primary care trusts between 2008 and 2009, and also reported a very weak association between CVI and score of Index of Multiple Deprivation (IMD) [R = 0.0329, *p* = 0.15], while Rahman et al. [6], interrogating CVI data in England and Wales reported a decrease in new certifications in 2017/2018 (41 per 100,000) compared 2010/2011 (43 per 100,000 population in 2010/11) mostly due to a reduction in certification due to AMD in people over the age of 65 years. They also reported a decrease in new certification due to diabetic eye disease in those aged 12+ years most likely due to improved uptake of national diabetic retinopathy screening and improved treatment for AMD. Evidence on local-level trends in CVI remains limited, hindering informed planning and the efficient allocation of resources.

Regular eye examinations may decrease the risk of sight impairment and CVI. Shickle et al. [14] explored geographical inequalities in the uptake of NHS funded eye examinations in Leeds using GOS-1 data, and in Essex [15], suggesting that those entitled to a free test in the *least* deprived areas were more likely to take up the free eye tests compared to those living in the most deprived areas. A recent study by Harper et al. [16] reported nearly three times lower density of optometry practices in more deprived areas compared to least deprived areas, and also nearly two times increased rate of practice closure in most deprived areas compared to the least deprived. Internationally, Shneor et al. [17] assessed the density of optometrists, mild to severe vision impairment and blindness in 34 Organization for Economic Cooperation and Development countries including the UK. They reported a significant inverse correlation between the density of optometrists and the prevalence of blindness [R = −0.40, *p* = 0.02].

Health inequalities are defined as avoidable, unfair, and systematic differences, influenced by various factors in addition to healthcare access such as socioeconomic factors (income), geography (urban or rural), sex, ethnicity, disability, and social exclusion (homelessness) [18]. To date, no study to our knowledge has explored possible associations between CVI and deprivation, number of primary care optometry practices, or age demographics of people in Mid and South Essex (MSE). We aim to do so for five sub-Integrated Care Board (ICB) locations (previously CCG areas) in MSE.

We hypothesised that CVI would be higher socio-economically deprived areas, lower optometry practice densities and where there are more older people. We utilised publicly available data on CVI, distribution of optometry practices and also age demographics to analyse trends and factors associated with CVI. We strategically prioritised the MSE ICB to provide a comprehensive assessment of the current landscape, with the aim of informing commissioning decisions to improve access to eye care services. The current MSE ICB (until April 2026), serves a population of 1.2 million people, previously under five CCG’s (Mid Essex, Thurrock, Basildon & Brentwood, Castle Point & Rochford and Southend) [19]. The main aim of this study is to explore, for each of the five sub-ICB locations, the number of CVI per 100,000, the rate of change of CVI over a two-year period (2021–2023), the percentage of people living in the 20% most deprived areas in each of the sub-locations, density of optometry practices and the percentage of people over the age of 60 years.

## Materials and methods

### Setting

The current MSE ICB operates within the East of England, overseeing healthcare services for a population of ~1.2 million people with the remit of delivering high-quality and best healthcare to improve people’s lives. Eye health care is commissioned across primary and secondary care settings in line with NHS service models in England. Primary eye care is largely delivered through community optometry practices, and patients requiring further investigation or treatment may be referred to specialist ophthalmology services in the region. Community diagnostic hubs have been established to reduce hospital waiting times by delivering care closer to patients’ homes. The MSE ICB is divided for data-monitoring purposes into five Sub-ICB locations (Sub-ICB-1, Sub-ICB-2, Sub-ICB-3, Sub-ICB-4, and Sub-ICB-5) [19, 20].

### Data sources

We conducted an analysis of retrospective anonymised data and publicly available data, and therefore ethical approval was not required. CVI data from 2021 to 2023 were obtained from the Royal College of Ophthalmologists’ certification office in Moorfields Eye Hospital. CVI data were available only for the year 2021–2023. Locations of primary care optometrists providing NHS services and the number of practices in each sub-ICB locations were derived from NHS Digital website [21]. The density of primary care optometry practices for 100,000 population for each of these five sub-ICB locations was then calculated using the population census data retrieved from the Office for National Statistics (ONS) [22].

Index of Multiple Deprivation (IMD) 2019 data was extracted from publicly available MSE Population Insight Pack-Version 2 [23]. We estimated the proportion of the population residing in the most deprived Lower-layer Super Output Areas within MSE sub-ICBs locations, as those falling within the first and second national deciles (0–10% and 10–20% most deprived) [22]. We opted for the most deprived 20% based on the IMD, aligning with the “CORE20PLUS5” framework of NHS England. The IMD 2019 is a composite measure of relative area-level deprivation in England, incorporating seven weighted domains: income; employment; education, skills and training; health and disability; crime; barriers to housing services; and living environment. The number of new CVI certifications per 100,000 population (2021–2023) were calculated using population census data for each sub-ICB for each year, and the rate of change of CVI was calculated to quantify the temporal trends. Population census data from ONS were extracted into Excel spreadsheets and summary tables were created for each sub-ICB locations [22]. Other variables, such as gender and ethnicity, were not available within the publicly accessible datasets used for this analysis.

### Statistical analysis

Descriptive statistics were summarised using Microsoft Excel (2024, Microsoft corporation). The rate of change in CVI between 2021 and 2023 was calculated as the proportional change in the number of CVIs over this period.

Associations between the rate of change of CVI over two years (2021–2023), and deprivation defined as the proportion of people living in the 20% most deprived areas within each sub-ICB, were explored, along with the density of primary care optometry practices at each sub-ICB level. Additionally, we examined associations between the CVI per 100,000 population in 2023, and both the density of optometry practices and the proportion of people aged more than 60 years in each sub-ICB. Due to small sample size and to avoid assumptions of normality, Spearman’s rank correlation (ρ) was used to estimate the strength and direction of associations, and 95% confidence intervals (CI) were calculated using bootstrap methods. Correlation coefficients were interpreted according to conventional thresholds (1 perfect; 0.9 to 0.7 strong; 0.6 to 0.4 moderate; 0.3 to 0.1 weak), and a *p* value < 0.05 was considered statistically significant. Statistical analyses were conducted using STATA-IC (Version 14.2, Corp LP, Texas, USA).

Results were reported based on the Strengthening the Reporting of Observational Studies in Epidemiology (STROBE) extended statement (RECORD) for reporting studies involving routinely collected health data (Supplementary File 1).

## Results

### CVI per 100,000 population

**Fig. 1:**
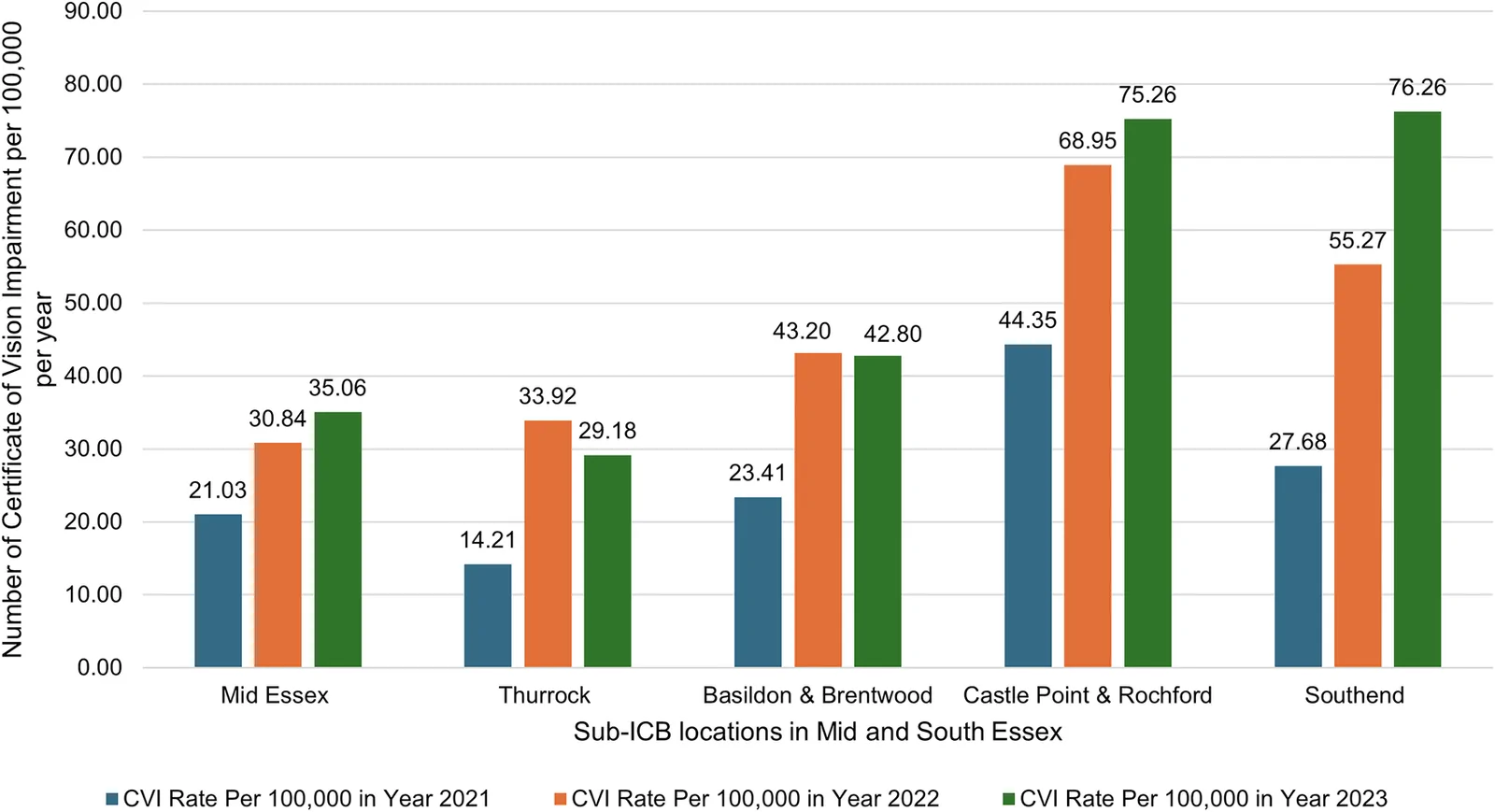
Certificate of Vision Impairment from 2021 to 2023 in the five sub-ICB locations in MSE.

Figure 1 shows CVI per 100,000 people living in the five sub-ICB locations in MSE between 2021 and 2023, showing 2.6-fold difference between the different ICB locations with Sub-ICB-2 showing the lowest recorded CVI (29.2 per 100,000) and Sub-ICB-5 showing the highest (76.3 per 100,000).

### Rate of change of CVI over the two-year period (2021–2023)

**Fig. 2:**
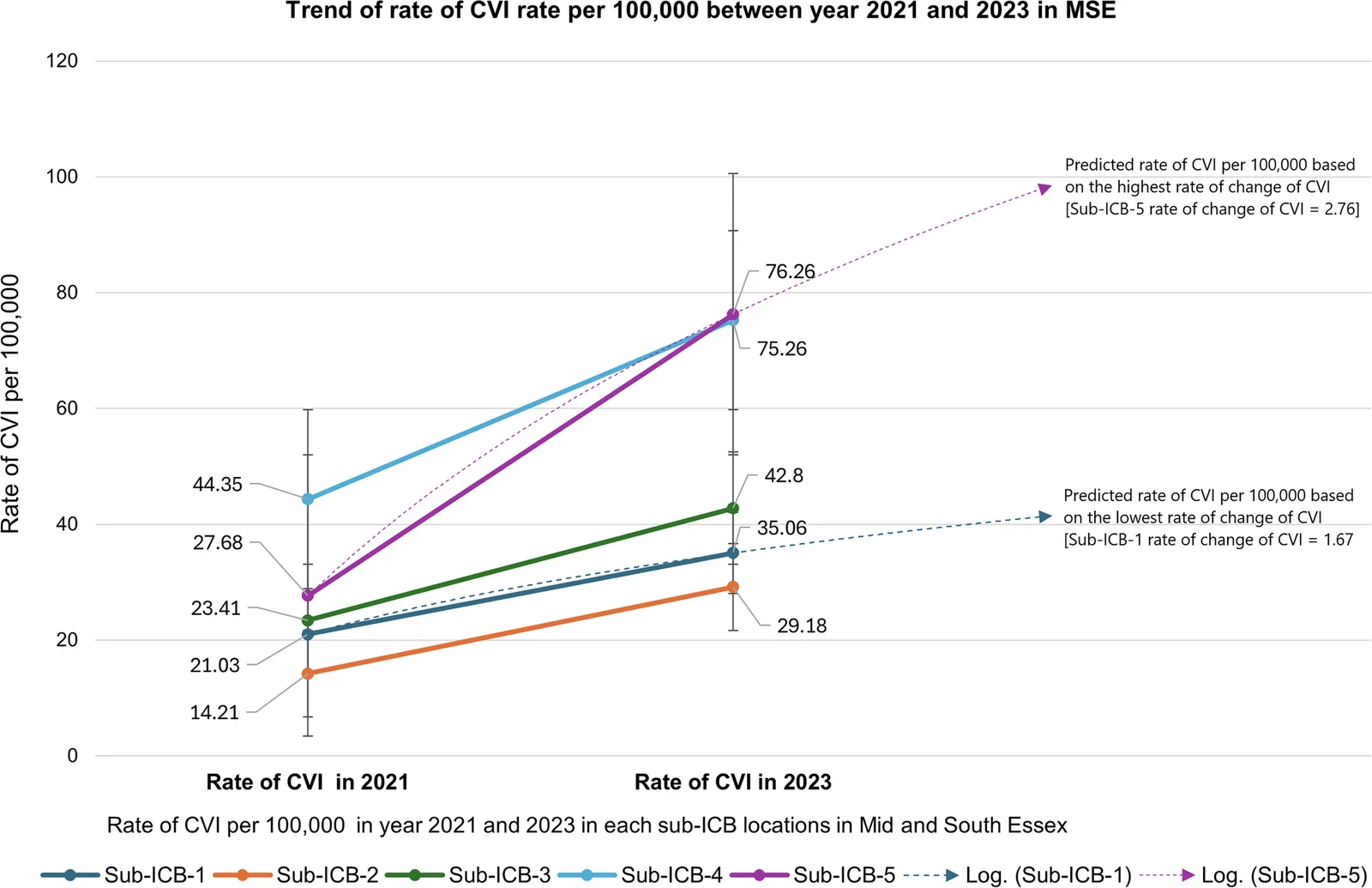
Temporal trend in rate of CVI per 100,000 between year 2021 and 2023 in MSE.

Figure 2 shows the rate of change of CVI: Sub-ICB-5 shows the highest increase of 2.76 times over the two years (2021–2023) and Sub-ICB-1 showing the lowest (1.67 times).

### Rate of change of CVI over two years (2021–2023) and deprivation in the five sub-ICB locations in MSE

**Fig. 3:**
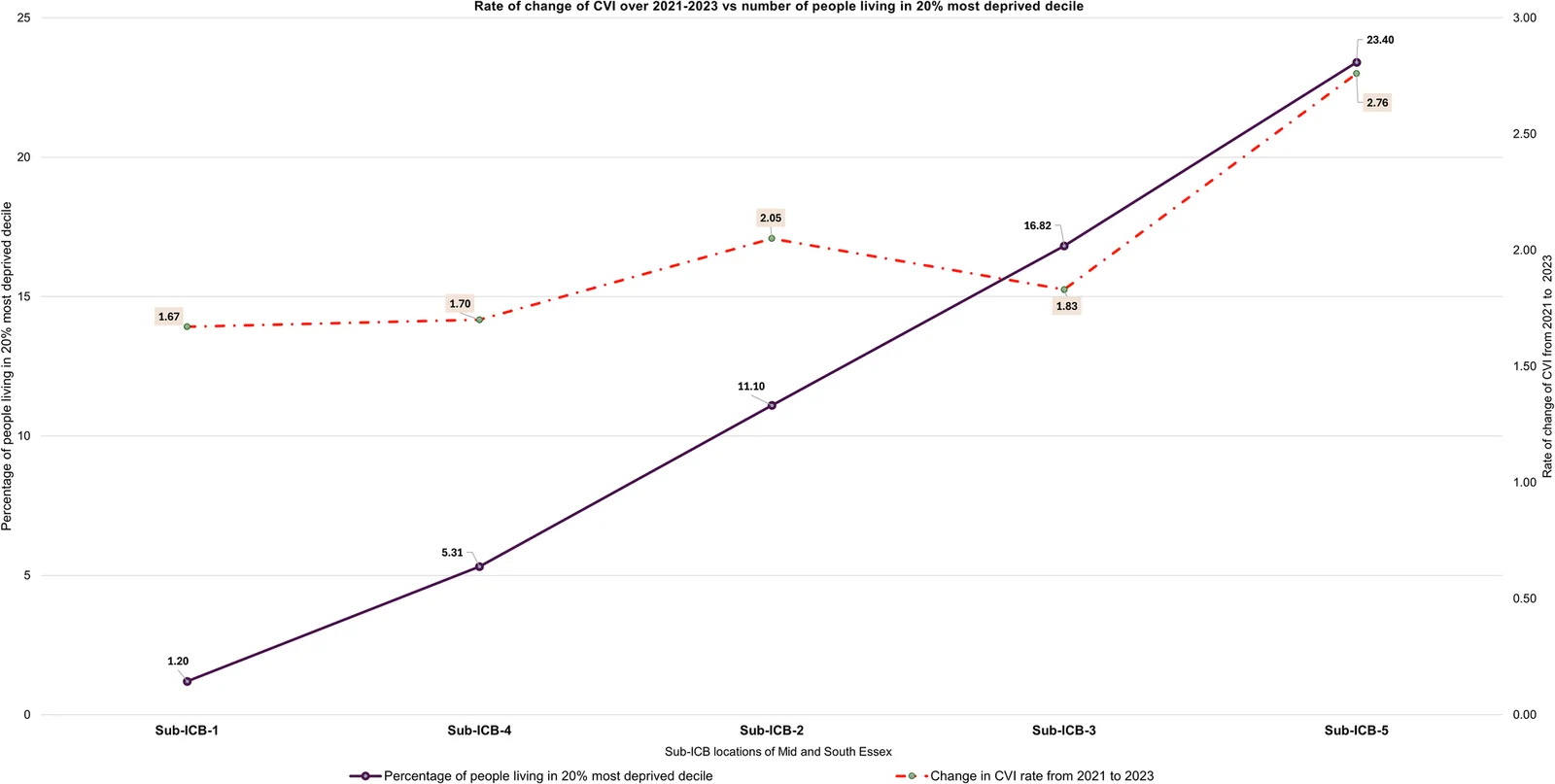
Rate of change of CVI over 2021–2023 compared with the percentage of people living in the 20% most deprived deciles in five sub-ICB locations of MSE.

Figure 3 shows rate of change of CVI per 100,000 people (2021–2023) plotted against the percentage of people living in the 20% most deprived deciles in the same sub-ICB locations. The number of CVI certifications per 100,000 people (2021–2023) and the percentage of people in the 20% most deprived decile are given in the Table 1. A strong positive association between the rate of change of CVI per 100,000 population (2021–2023) and deprivation (Spearman’s ρ = 0.90, 95% CI 0.35–1.00, *p* = 0.0374) was shown.

**Table 1:** Number of CVI certifications per 100,000 and percentage of people in 20% most deprived decile of each sub-ICB location of MSE.

Sub-ICB region of MSE	CVI certifications per 100,000 in year 2021	CVI certifications per 100,000 in year 2022	CVI certifications per 100,000 in year 2023	Percentage of people in 20% most deprived decile	Proportion of people over 60 years	Density of optometry practices (per 100,000 people)
Sub-ICB-1	21.03	30.84	35.06	1.20%	27.00%	9.55
Sub-ICB-2	14.21	33.92	29.18	11.10%	19.00%	11.31
Sub-ICB-3	23.41	43.20	43.80	16.82%	24.00%	11.27
Sub-ICB-4	44.35	68.95	75.26	5.31%	31.00%	18.65
Sub-ICB-5	27.68	55.27	76.26	23.40%	25.00%	17.14

Sub ICB-5 shows the highest proportion of people living in the 20% deprived areas and also has the highest number of CVI. Interestingly Sub ICB-4, which also has one of the highest rates of CVI (75.26 per 100,000 in year 2023) is not located in an area of deprivation suggesting that other factors also contribute including possibly access to transport etc.

### Density of primary care optometry practices and CVI per 100,000 people in the five sub-ICB locations in MSE

**Fig. 4:**
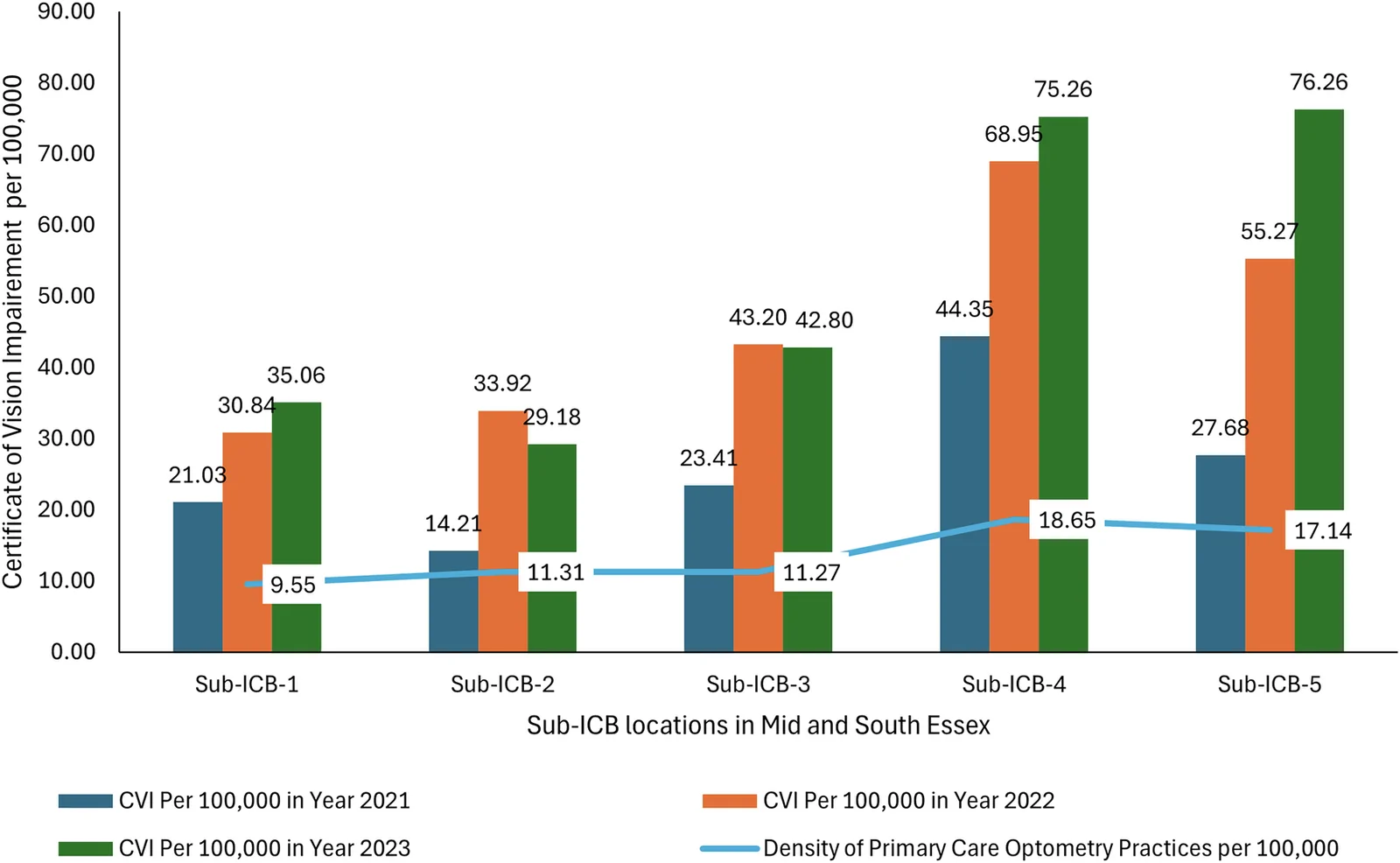
CVI per 100,000 in MSE from 2021 to 2023 and the density of primary care optometry practices.

Figure 4 shows CVI per 100,000 people and the density of primary care optometry practices in different sub-ICB areas. Both Sub-ICB-4 and Sub-ICB-5 recorded the highest number of CVI of 76.3/100,000 but record high primary care optometry practice densities (Sub-ICB-5 17.1/100,000; Sub-ICB-4 18.6/100,000). A moderate positive association was observed between the rate of CVI per 100,000 population in 2023 and the density of primary care optometry practices (Spearman’s ρ = 0.60, 95% CI −0.27 to 1.00, *p* = 0.285) reflecting higher demand for services in the areas with higher rates of CVI.

### CVI and the percentage of people over the age of 60 years in different sub-ICB locations in MSE

Sub-ICB-4 had the highest percentage of older people ≥60 years (31%) and had one of the highest CVI in 2023. A moderate positive association was observed between CVI in year 2023 and the percentage of people over the age of 60 years (Spearman’s ρ = 0.50, 95% CI −0.52 to 1.00, *p* = 0.391). Although Sub-ICB-5 recorded the highest CVI in 2023, it does not have the highest number of people over the age of 60 years, but it has the highest number of people living in 20% deprived areas (see Supplementary File 2 - Fig. 1).

### Causes of CVI in MSE over 2021–2023

Supplementary File 2 - Fig. 2 shows the causes of CVI in each sub-ICB of MSE over the two-year period (2021–2023). The most common cause of CVI was AMD (incident cases of *n* = 726 persons from 2021 to 2023), with the highest number shown in Sub-ICB-4 (*n* = 206) followed by ‘other causes’ (stroke, optic atrophy, visual cortex disorders, hereditary eye disease; incident cases of *n* = 395 from 2021 to 2023). CVI due to diabetic retinopathy also increased, possibly due to reduced screening uptake during the COVID pandemic and Sub-ICB-1 had highest number of incident cases of CVI due to diabetic retinopathy (*n* = 11 persons over from 2021 to 2023). Over the period of 2021 to 2023 in MSE, CVI due to AMD increased from 12.8/100,000 (2021), to 23.4/100,000 (2023), while ‘other causes’ increased 5.96/100,000 to 13.8/100,000 in the same period.

## Discussion

Our study is the first study to report on the levels and factors that might affect CVI in MSE. We found an overall increase in the rate of CVI change over the period of 2021–2023 in the different sub-ICB locations of MSE and also geographical variations in new registration of CVI per 100,000. Statistically significant strong association was observed between rate of change of CVI and deprivation and demonstrating an association between socio-economic factors and vision impairment. Sub-ICB-5, which had the highest CVI rate change also had the largest percentage of people living in the 20% most deprived deciles, while Sub-ICB-1 with the lowest proportion of people in the 20% most deprived decile also the lowest CVI. This agrees with a recent study in Northern Ireland found that individuals living in more deprived areas were significantly (80.97 vs 85.77; *p* < 0.001) more likely to be certified as CVI [24].

A systematic review identified several social determinants that were inversely associated with vision impairment including higher socio-economic status, higher education status and non-manual occupations [25]. Studies in the UK have shown correlations between socio-economic deprivation and the occurrence of more advanced vision field loss in glaucoma [26], lower attendance in diabetic eye screening and higher rates of sight-threatening retinopathy diagnosis [27–30] and an increased odds of presenting with severe visual acuity reduction due to AMD compared with residing in less deprived areas (odds ratio 3.59; *p* = 0.01) [31]. A study from Birmingham (UK) reported that patients with neovascular AMD in greater social deprivation areas had the worse visual acuity (correlation 0.185; *p* = 0.043) at the first visit with earlier CVI registration [32]. Data analysis from UK biobank suggests that individuals reporting glaucoma had more adverse socio-economic characteristics (Townsend deprivation index −0.72; *p* < 0.001) [33]. Our study findings, with other studies support the implementation of targeted early screening among socio-economically deprived populations to facilitate earlier detection and intervention.

Moderate positive associations were shown between CVI per 100,000 and older age profile. It is noteworthy that Sub-ICB-4 exhibited a high CVI despite having a relatively low percentage of the population living in deprived areas of MSE; likely due to the higher percentage of older people (>60 years) living there. AMD is generally an older person’s disease, so it is not surprising that this effect was shown. Targeted interventions focusing on both deprived communities and older populations would help reduce the burden of vision impairment in MSE. A larger general practice-based study conducted in the UK reported increasing prevalence of low vision among the people aged 75–79 (5.6%) and 90+ (30.0%) compatible with observations we made for MSE [34].

In contrast to the previous studies [16, 17], CVI rates in MSE do not appear to be statistically significantly associated with the density of primary care optometrist practices. Sub-ICB-4 and Sub-ICB-5 show the highest levels of CVI and the highest densities of primary care optometry practices. Whether this is due to higher rate of detection and higher number of referrals from primary to secondary care, with a corresponding increase in demand for timely care and prevention of disease progression needs to be ascertained.

### Strengths and limitations of the study

This study leverages fairly accurate and comprehensive CVI data available for 2021–2023 and represents probably the first investigation of these associations in MSE, thereby filling an important gap in literature. The findings provide valuable insights to improve eye health services organisation and resource allocation in the MSE, targeting deprived areas and those with higher populations of older people.

There are some limitations of this study that could not be mitigated. The latest index of publicly available data on multiple deprivation was from 2019, which may not reflect the levels of deprivation for the time period covered by the CVI data. In addition, there may be under reporting of the CVI data across different sub-ICB locations; however, care was taken to ensure that data obtained from the central repository of Moorfields Eye Hospital were used accurately. In addition, it would have been useful to have access to more granular data, including gender, age at CVI certification, and ethnic group, but these were not available. Another limitation of the current analysis is its reliance on CVI data from the year 2023, which may not fully reflect the trends observed in MSE. Notably, the sub-ICB-2 region exhibited a higher number of CVIs in 2022 compared to 2023, suggesting potential temporal variability may not be fully reflected in the current analysis. The robustness of the cross-sectional associations described in this study could be enhanced by conducting longitudinal analyses, which may require individual-level participant data coupled with their geographical locations in cohort study designs. Such granular data integration in a larger sample may allow for a deeper exploration of the associations observed in the current study.

### Conclusions and recommendations

This analysis highlights strong positive association between higher rates of CVI and the proportion of people living in the 20% most deprived areas. Moderate positive associations were observed between CVI per 100,000 population and the proportion of people over the age of 60, as well as between CVI per 100,000 and the density of primary care optometry practices across the different locations of MSE sub-ICBs. This study suggests that areas with a higher percentage of people living in deprived areas, and areas that had a higher percentage of people over the age of 60 years reported higher levels of CVI.

The most common cause of CVI was AMD. There was also an increase in CVI due to diabetic retinopathy, which may be reflection of the COVID-19 time period. Our findings highlight the need for targeted interventions to reduce vision impairment, particularly focusing on communities living in deprived areas and on older populations. Addressing the root causes of deprivation, improving access to eye care through proactive screening in areas where deprivation and older age populations are co-located, and raising awareness about preventable conditions such as AMD and diabetic retinopathy should be key priorities in reducing the overall burden of vision loss. Access, long waiting lists, and the validity of CVI data recording should be explored in future studies.

## Summary

### What was known before

- Geographical variations in rates of Certificate of Vision Impairment (CVI) have been studied in the UK, however the associations between CVI, deprivation and other indicators such as density of optometry practices have not been evaluated.

### What this study adds

- Our study highlights associations between CVI, the number of people living in the 20% most deprived areas and proportion of older people.
- Our findings highlight the need to develop targeted interventions to reduce vision impairment, particularly focusing on older populations and communities living in deprived areas.

## Supplementary information

## Data Availability

The data that support the findings of this study are available from the corresponding author upon reasonable request.
