# Supplementary material for: Exploring variations in Certificate of Vision Impairment (CVI) with geographical locations, deprivation indices, density of optometry practices and number of older people living in Mid and South Essex: an analysis of publicly available data
Source: Eye (Lond). 2026 Jun 27;40(13):2093–9. doi: 10.1038/s41433-026-04627-6 (PMC13538598; doi:10.1038/s41433-026-04627-6)
Supplement: Supplementary file 2 [file 41433_2026_4627_MOESM1_ESM.docx]

**Study Title - Exploring rates of Certificate of Vision Impairment (CVI) with geographical locations, deprivation indices, density of optometry practices and number of older people living in Mid and South Essex: An analysis of publicly available data**

**Supplementary Files**

**Supplementary File 1: The RECORD statement – checklist of items, extended from the STROBE statement, that should be reported in observational studies using routinely collected health data.**

|  | **Item No.** | **STROBE items** | **Location in manuscript where items are reported** | **RECORD items** | **Location in manuscript where items are reported** |
| --- | --- | --- | --- | --- | --- |
| **Title and abstract** | | | | | |
|  | 1 | (a) Indicate the study’s design with a commonly used term in the title or the abstract (b) Provide in the abstract an informative and balanced summary of what was done and what was found | Page No 1 and 5-6 | RECORD 1.1: The type of data used should be specified in the title or abstract. When possible, the name of the databases used should be included.  RECORD 1.2: If applicable, the geographic region and timeframe within which the study took place should be reported in the title or abstract.  RECORD 1.3: If linkage between databases was conducted for the study, this should be clearly stated in the title or abstract. | Page No 1 and 4-5 |
| **Introduction** | | | | | |
| Background rationale | 2 | Explain the scientific background and rationale for the investigation being reported | Page No 7-10 |  |  |
| Objectives | 3 | State specific objectives, including any prespecified hypotheses | Page 10 |  |  |
| **Methods** | | | | | |
| Study Design | 4 | Present key elements of study design early in the paper | Page 11 |  |  |
| Setting | 5 | Describe the setting, locations, and relevant dates, including periods of recruitment, exposure, follow-up, and data collection | Page 10-11 |  |  |
| Participants | 6 | *(a) Cohort study* - Give the eligibility criteria, and the sources and methods of selection of participants. Describe methods of follow-up  *Case-control study* - Give the eligibility criteria, and the sources and methods of case ascertainment and control selection. Give the rationale for the choice of cases and controls  *Cross-sectional study* - Give the eligibility criteria, and the sources and methods of selection of participants  *(b) Cohort study* - For matched studies, give matching criteria and number of exposed and unexposed  *Case-control study* - For matched studies, give matching criteria and the number of controls per case | Page 11 | RECORD 6.1: The methods of study population selection (such as codes or algorithms used to identify subjects) should be listed in detail. If this is not possible, an explanation should be provided.  RECORD 6.2: Any validation studies of the codes or algorithms used to select the population should be referenced. If validation was conducted for this study and not published elsewhere, detailed methods and results should be provided.  RECORD 6.3: If the study involved linkage of databases, consider use of a flow diagram or other graphical display to demonstrate the data linkage process, including the number of individuals with linked data at each stage. | Not Applicable  Not Applicable  Not Applicable |
| Variables | 7 | Clearly define all outcomes, exposures, predictors, potential confounders, and effect modifiers. Give diagnostic criteria, if applicable. | Page 11-12 | RECORD 7.1: A complete list of codes and algorithms used to classify exposures, outcomes, confounders, and effect modifiers should be provided. If these cannot be reported, an explanation should be provided. | Not Applicable |
| Data sources/ measurement | 8 | For each variable of interest, give sources of data and details of methods of assessment (measurement).  Describe comparability of assessment methods if there is more than one group | Page 11-12 |  |  |
| Bias | 9 | Describe any efforts to address potential sources of bias | Not Applicable |  |  |
| Study size | 10 | Explain how the study size was arrived at | Not Applicable |  |  |
| Quantitative variables | 11 | Explain how quantitative variables were handled in the analyses. If applicable, describe which groupings were chosen, and why | Page 11-12 |  |  |
| Statistical methods | 12 | (a) Describe all statistical methods, including those used to control for confounding  (b) Describe any methods used to examine subgroups and interactions  (c) Explain how missing data were addressed  (d) *Cohort study* - If applicable, explain how loss to follow-up was addressed  *Case-control study* - If applicable, explain how matching of cases and controls was addressed  *Cross-sectional study* - If applicable, describe analytical methods taking account of sampling strategy  (e) Describe any sensitivity analyses | Page 11,12 |  |  |
| Data access and cleaning methods |  | .. |  | RECORD 12.1: Authors should describe the extent to which the investigators had access to the database population used to create the study population.  RECORD 12.2: Authors should provide information on the data cleaning methods used in the study. | Not Applicable |
| Linkage |  | .. |  | RECORD 12.3: State whether the study included person-level, institutional-level, or other data linkage across two or more databases. The methods of linkage and methods of linkage quality evaluation should be provided. | Not Applicable |
| **Results** | | | | | |
| Participants | 13 | (a) Report the numbers of individuals at each stage of the study (*e.g.*, numbers potentially eligible, examined for eligibility, confirmed eligible, included in the study, completing follow-up, and analysed)  (b) Give reasons for non-participation at each stage.  (c) Consider use of a flow diagram | Page 13-14 | RECORD 13.1: Describe in detail the selection of the persons included in the study (*i.e.,* study population selection) including filtering based on data quality, data availability and linkage. The selection of included persons can be described in the text and/or by means of the study flow diagram. | Not Applicable |
| Descriptive data | 14 | (a) Give characteristics of study participants (*e.g.*, demographic, clinical, social) and information on exposures and potential confounders  (b) Indicate the number of participants with missing data for each variable of interest  (c) *Cohort study* - summarise follow-up time (*e.g.*, average and total amount) | Not Applicable |  |  |
| Outcome data | 15 | *Cohort study* - Report numbers of outcome events or summary measures over time  *Case-control study* - Report numbers in each exposure category, or summary measures of exposure  *Cross-sectional study* - Report numbers of outcome events or summary measures | Page No 13-15 |  |  |
| Main results | 16 | (a) Give unadjusted estimates and, if applicable, confounder-adjusted estimates and their precision (e.g., 95% confidence interval). Make clear which confounders were adjusted for and why they were included  (b) Report category boundaries when continuous variables were categorized  (c) If relevant, consider translating estimates of relative risk into absolute risk for a meaningful time period | Not Applicable |  |  |
| Other analyses | 17 | Report other analyses done—e.g., analyses of subgroups and interactions, and sensitivity analyses | Page No 15-16 |  |  |
| **Discussion** | | | | | |
| Key results | 18 | Summarise key results with reference to study objectives | Page No 16 and 19 |  |  |
| Limitations | 19 | Discuss limitations of the study, taking into account sources of potential bias or imprecision. Discuss both direction and magnitude of any potential bias | Page No 18-19 | RECORD 19.1: Discuss the implications of using data that were not created or collected to answer the specific research question(s). Include discussion of misclassification bias, unmeasured confounding, missing data, and changing eligibility over time, as they pertain to the study being reported. | Page No 18-19 |
| Interpretation | 20 | Give a cautious overall interpretation of results considering objectives, limitations, multiplicity of analyses, results from similar studies, and other relevant evidence | Page No 19 |  |  |
| Generalisability | 21 | Discuss the generalisability (external validity) of the study results | Page No 19-20 |  |  |
| **Other Information** | | | | | |
| Funding | 22 | Give the source of funding and the role of the funders for the present study and, if applicable, for the original study on which the present article is based | Page No 21 |  |  |
| Accessibility of protocol, raw data, and programming code |  | .. | Page No 21 | RECORD 22.1: Authors should provide information on how to access any supplemental information such as the study protocol, raw data, or programming code. | Page No 21-22 |

**Supplementary File 2 - Figure 1:**

CVI per 100,000 in year 2023 vs percentage of people > 60 years old

| Percentage of people > 60 years | CVI per 100,000 in 2023 |
| --- | --- |
| 27 | 35.06 |
| 19 | 29.18 |
| 24 | 42.8 |
| 31 | 75.26 |
| 25 | 76.26 |

**Figure 1.** CVI per 100,000 in 2023 and percentage of people > 60 years of age

**Supplementary File 2 - Figure 2:**

Causes of CVI in each Sub-ICB location of MSE in year 2021-2023

Number of people with each eye disease at each sub-ICB location in MSE

| Sub-ICB location of MSE and year | Cause of CVI_DR | Cause of CVI_Glaucoma | Cause of CVI_Other# | Cause of CVI_AMD |
| --- | --- | --- | --- | --- |
| 2020/21 Sub-ICB-2 | 0 | 0 | 0 | 12 |
| 2020/21 Sub-ICB-5 | 0 | 0 | 14 | 27 |
| 2020/21 Sub-ICB-1 | 0 | 10 | 39 | 34 |
| 2020/21 Sub-ICB-3 | 0 | 6 | 6 | 39 |
| 2020/21 Sub-ICB-4 | 0 | 7 | 15 | 47 |
| 2021/22 Sub-ICB-2 | 0 | 0 | 13 | 35 |
| 2021/22 Sub-ICB-1 | 6 | 12 | 60 | 44 |
| 2021/22 Sub-ICB-5 | 0 | 8 | 20 | 56 |
| 2021/22 Sub-ICB-3 | 7 | 13 | 31 | 58 |
| 2021/22 Sub-ICB-4 | 0 | 10 | 25 | 82 |
| 2022/23 Sub-ICB-2 | 0 | 6 | 7 | 26 |
| 2022/23 Sub-ICB-3 | 0 | 18 | 36 | 53 |
| 2022/23 Sub-ICB-1 | 5 | 13 | 57 | 64 |
| 2022/23 Sub-ICB-5 | 5 | 16 | 39 | 72 |
| 2022/23 Sub-ICB-4 | 6 | 10 | 33 | 77 |

**Figure 2.** Causes of CVI over the period of 2021 to 2023 in each sub-ICB of MSE

*(#Other causes of CVI includes: Stroke, Optic atrophy, Visual cortex disorders, Hereditary eye disease, Multiple and Other causes unclassified in the database; *Missing data: When the number is less than 5 persons for any eye disease/condition it is not shown in the data shared by the Moorfields Eye Hospital)*
